# New insights into meningitic *Escherichia coli* infection of brain microvascular endothelial cells from quantitative proteomics analysis

**DOI:** 10.1186/s12974-018-1325-z

**Published:** 2018-10-19

**Authors:** Wen-Tong Liu, Yu-Jin Lv, Rui-Cheng Yang, Ji-Yang Fu, Lu Liu, Huan Wang, Qi Cao, Chen Tan, Huan-Chun Chen, Xiang-Ru Wang

**Affiliations:** 1 The Cooperative Innovation Center for Sustainable Pig Production, Huazhong Agricultural University, Wuhan, 430070 Hubei China; 2 College of Veterinary Medicine, Henan University of Animal Husbandry and Economy, Zhengzhou, 450046 Henan China; 3 State Key Laboratory of Agricultural Microbiology, College of Veterinary Medicine, Huazhong Agricultural University, Wuhan, 430070 Hubei China

**Keywords:** iTRAQ, Proteomics, Blood-brain barrier, BMECs, Meningitic *E. coli*

## Abstract

**Background:**

Bacterial meningitis remains a big threat to the integrity of the central nervous system (CNS), despite the advancements in antimicrobial reagents. *Escherichia coli* is a bacterial pathogen that can disrupt the CNS function, especially in neonates. *E. coli* meningitis occurs after bacteria invade the brain microvascular endothelial cells (BMECs) that form a direct and essential barrier restricting the entry of circulating microbes and toxins to the brain. Previous studies have reported on several cellular proteins that function during meningitic *E. coli* infections; however, more comprehensive investigations to elucidate the potential targets involved in *E. coli* meningitis are essential to better understand this disease and discover new treatments for it.

**Methods:**

The isobaric tags for relative and absolute quantification (iTRAQ) approach coupled with LC-MS/MS were applied to compare and characterize the different proteomic profiles of BMECs in response to meningitic or non-meningitic *E. coli* strains. KEGG and gene ontology annotations, ingenuity pathways analysis, and functional experiments were combined to identify the key host molecules involved in the meningitic *E. coli*-induced tight junction breakdown and neuroinflammatory responses.

**Results:**

A total of 13 cellular proteins were found to be differentially expressed by meningitic *E. coli* strains PCN033 and RS218, including one that was also affected by HB101, a non-meningitic *E. coli* strain. Through bioinformatics analysis, we identified the macrophage migration inhibitory factor (MIF), granzyme A, NF-κB signaling, and mitogen-activated protein kinase (MAPK) pathways as being biologically involved in the meningitic *E. coli*-induced tight junction breakdown and neuroinflammation. Functionally, we showed that MIF facilitated meningitic *E. coli*-induced production of cytokines and chemokines and also helped to disrupt the blood-brain barrier by decreasing the expression of tight junction proteins like ZO-1, occludin. Moreover, we demonstrated the significant activation of NF-κB and MAPK signaling in BMECs in response to meningitic *E. coli* strains, which dominantly determined the generation of the proinflammatory cytokines including IL-6, IL-8, TNF-α, and IL-1β.

**Conclusions:**

Our work identified 12 host cellular targets that are affected by meningitic *E. coli* strains and revealed MIF to be an important contributor to meningitic *E. coli*-induced cytokine production and tight junction disruption, and also the NF-κB and MAPK signaling pathways that are mainly involved in the infection-induced cytokines production. Characterization of these distinct proteins and pathways in BMECs will facilitate further elucidation of meningitis-causing mechanisms in humans and animals, thereby enabling the development of novel preventative and therapeutic strategies against infection with meningitic *E. coli*.

**Electronic supplementary material:**

The online version of this article (10.1186/s12974-018-1325-z) contains supplementary material, which is available to authorized users.

## Background

Bacterial meningitis is a severe, life-threatening infection of the central nervous system (CNS) with high morbidity and mortality. It is currently recognized as one of the top ten killers in infection-related deaths worldwide, with almost half of the survivors suffering from diverse neurological sequelae (e.g., mental retardation, hearing impairment and blindness), despite the advancements made in the field of antimicrobial treatment [1–3]. Most bacterial meningitis cases are initiated by hematogenous spread and develop when the circulating bacteria penetrate the blood-brain barrier (BBB), destroy brain parenchyma, and finally cause CNS disorders [1]. Among the meningitis-causing microbes, extraintestinal pathogenic *Escherichia coli* (ExPEC) has recently emerged as an important zoonotic bacterial pathogen with the potential to colonize multiple tissues outside the intestine and cause severe infections, with one typical outcome being meningitis. The evidence from recent in vivo and in vitro studies indicates that meningitic *E. coli* strains possess the ability to invade the brain, and the infection-induced BBB disruption that occurs is the hallmark event in the development of *E. coli* meningitis [4, 5].

The availability of in vitro and in vivo BBB infection models has made the study of meningitic *E. coli* penetration of the brain possible [6–9]. The in vitro BBB model uses brain microvascular endothelial cells (BMECs) that form distinctive tight junctions and exhibit high trans-endothelial electrical resistance, thereby mimicking the features of the natural in vivo barrier that protects the brain from circulating microorganisms and toxins [10–13]. The in vivo model is established by inducing experimental hematogenous meningitis in newborn rats and mice [9, 14, 15]. With these models, it is now well-established that successful traversal of the BBB by circulating *E. coli* strains requires the following prerequisites: a high bacteremia, binding to and invasion of BMECs, rearrangement of actin cytoskeleton, and crossing the BBB as live bacteria [1, 2]. These require a series of complicated interactions between meningitic *E. coli* and the host. So far, several host targets have been found to be associated with this invasion process, including certain intracellular signaling molecules like focal adhesion kinase, phosphatidylinositol 3-kinase (PI3K), Rho GTPases, cytosolic phospholipase A2, nuclear factor-κB (NF-κB), inducible nitric oxide synthase (NOS), and several cellular surface molecules/receptors such as caveolin-1, Toll-like receptors, the intercellular adhesion molecule (ICAM-1), and some actin-binding molecules like ERM family proteins (ezrin, radixin, and moesin), most likely through their influences on the aforementioned prerequisites [8, 16–19]. We have previously identified and characterized two essential cellular targets, S1P and EGFR, which are exploited by meningitic *E. coli* for successful invasion of the BBB [20]. In other work, we have also found that vascular endothelial growth factor A (VEGFA) and Snail-1, which are inducible by meningitic *E. coli*, can mediate the BBB disruption [5]. Despite these advances, the mechanisms involved in CNS infection by meningitic *E. coli* are still poorly understood, and a more comprehensive investigation to elucidate the cellular targets in infected BMECs is now required.

In the current study, we compared the different proteomic profiles of BMECs in response to meningitic and non-meningitic *E. coli* strains via the isobaric tags for relative and absolute quantification (iTRAQ) approach and investigated the potential host factors and mechanisms that were hijacked by meningitic *E. coli* to penetrate the BBB. Characterization of these potential host targets will expand our current knowledge on meningitic *E. coli*-induced CNS infections and provide new strategies to prevent this infection and develop novel therapeutic reagents against it.

## Methods

### Bacterial strains, cell culture, and infection

The *E. coli* K1 strain RS218 (O18:K1:H7) [GenBank: CP007149.1], whose genomic sequencing has been finalized and annotated, is a well-characterized cerebrospinal fluid (CSF) isolate from a neonatal meningitis case [21]. The porcine-originated ExPEC strain PCN033 (O11: K2) [GenBank: CP006632.1], which was isolated from swine CSF in China [22, 23], is evidenced to be highly virulent and capable of invading and disrupting the BBB, thereby causing CNS dysfunction [5, 24]. *E. coli* K12 strain HB101 is an avirulent and non-meningitic strain normally used as a negative control strain [25, 26]. All *E. coli* strains were grown aerobically at 37 °C in Luria–Bertani medium unless otherwise specified.

The immortalized human BMECs (hereafter called hBMECs) were kindly provided by Prof. Kwang Sik Kim in Johns Hopkins University School of Medicine and routinely cultured in RPMI 1640 supplemented with 10% fetal bovine serum, 2 mM L-glutamine, 1 mM sodium pyruvate, essential amino acids, nonessential amino acids, vitamins, and penicillin and streptomycin (100 U/mL) in a 37 °C incubator under 5% CO_2_ until monolayer confluence was reached [20, 27]. Confluent cells were washed with Hank’s balanced salt solution (Corning Cellgro, Manassas, VA, USA) and starved in serum-free medium for 16–18 h before further treatment. For bacterial challenge, the cells were infected with *E. coli* PCN033, RS218, or HB101 strains each at a multiplicity of infection of 10 for 2 h. In some assays, the cells were pretreated with specific inhibitors prior to bacterial challenge.

### Reagents, antibodies, and inhibitors

The p38 inhibitor SB202190, extracellular signal-regulated kinases 1 and 2 (ERK1/2) inhibitor U0126, c-Jun N-terminal kinase (JNK) inhibitor SP600125, NF-κB inhibitor BAY11-7082, and (S, R)-3-(4-hydroxyphenyl)-4, 5-dihydro-5-isoxazole acetic acid methyl ester (ISO-1), an inhibitor of macrophage migration inhibitory factor (MIF), were purchased from MedChem Express (Monmouth, NJ, USA). Recombinant MIF protein was purchased from Novoprotein (Summit, NJ, USA). The nucleic acid dye, 4′-6-diamidino-2-phenylindole (DAPI), was obtained from Solarbio (Beijing, China). Anti-ZO-1, anti-MIF, anti-TATA box-binding protein-like protein 1 (TBPL1), anti-legumain (LGMN), anti-ERK1/2, and anti-phospho-ERK1/2 antibodies (all rabbit) were purchased from ABclonal (Wuhan, Hubei, China). Anti-occludin, anti-dystrophin (DMD), anti-HISTIHIC, anti-JNK, and anti-p38 mitogen-activated protein kinase (MAPK) antibodies (all rabbit) were purchased from Proteintech (Chicago, IL, USA). Anti-phospho-JNK (rabbit) antibody was from R&D Systems (Minneapolis, MO, USA). Anti-phospho-p38, anti-p65, anti-phospho-p65, and anti-IκBα antibodies (all rabbit) were purchased from Cell Signaling Technology (Danvers, MA, USA). Cy3-labeled goat anti-rabbit antibody was purchased from Beyotime Institute of Biotechnology (Shanghai, China). Anti-GAPDH (mouse) antibody was purchased from Beijing Biodragon Immunotechnologies Co., Ltd. (Beijing, China).

### Protein isolation, digestion, and labeling with iTRAQ reagents

Bacterial-infected and non-infected cells in 10 cm dishes were collected 2-h post-infection and gently washed with pre-chilled PBS buffer. The cells were lysed in 1 mL lysis buffer, and the soluble protein fraction was harvested by 5 min of ultrasonication treatment (pulse on 2 s, pulse off 3 s, power 180 W) followed by centrifugation at 20000×*g* for 30 min at 4 °C, and the protein concentration was determined via the Bradford protein assay method with BSA as the standard substance. The proteins were reduced with 10 mM iodoacetamide at room temperature for 45 min in the dark and then precipitated in acetone at − 20 °C for 3 h. After centrifugation at 20000×*g* for 20 min, the protein pellet was resuspended and ultrasonicated in pre-chilled 50% (*w*/*v*) tetraethyl-ammonium bromide (TEAB) buffer supplemented with 0.1% SDS. The proteins were obtained after centrifugation at 20000×*g* and their concentrations were measured by Bradford assays.

Subsequently, protein (100 μg) in TEAB buffer was incubated with 3.3 μL of trypsin (1 μg/μL) (Promega, Madison, WI, USA) at 37 °C for 24 h in a sealed tube. The tryptic peptides were lyophilized and dissolved in 50% TEAB buffer, and iTRAQ labeling was performed according to the manufacturer’s instructions (AB Sciex, Foster City, CA, USA). Briefly, one unit of iTRAQ reagent was thawed and reconstituted in 24 μL isopropanol and the peptides were incubated at room temperature for 2 h. The peptides from the control, HB101, PCN033, and RS218 groups were designated 114, 115, 116, and 117, respectively. The labeled samples were then mixed and dried with a rotary vacuum concentrator. The labeling efficiency was examined by mass spectrometry (MS).

### Strong cation exchange chromatography (SCX) fractionation and liquid chromatography (LC)–MS/MS analysis

The labeled samples were pooled and purified using an SCX column (Phenomenex, USA), and separated by LC using an LC-20AB HPLC pump system (Shimadzu, Japan). The peptides were then mixed with nine times their volume in buffer A (25% ACN, 10 mM KH_2_PO_4_, pH = 3) and loaded onto a 4.6 × 250 mm Ultremex SCX column containing 5-μm particles (Phenomenex). The peptides were eluted at a flow rate of 1 ml/min in a buffer B (25% ACN, 2 M KCL, 10 mM KH_2_PO_4_, pH = 3) gradient as follows: 0–5% buffer B for 30 min, 5–30% buffer B for 20 min, 30–50% buffer B for 5 min, 50% buffer B for 5 min, 50–100% buffer B for 5 min, and 100% buffer B for 1 min before equilibrating with buffer A for 10 min prior to the next injection. Next, the eluted peptides were desalted with a Strata X C18 column (100 mm × 75 mm, 5-um particles, 300A aperture) (Phenomenex, Torrance, CA, USA) and vacuum dried. The fractions were then dissolved in aqueous solution containing 0.1% formic acid (FA) and 2% ACN and centrifuged at 12000*g* for 10 min at 4 °C. Five micrograms supernatant was loaded on an LC-20AD nano HPLC (Shimadzu, Kyoto, Japan) by the autosampler onto a 2 cm C18 trap column (inner diameter 200 μm, Waters), and the peptides were eluted onto a resolving 10 cm analytical C18 column (inner diameter 75 μm, Waters). The mobile phases used were composed of solvent A (0.1% FA and 5% ACN) and solvent B (0.1% FA and 95% ACN). The gradient was run at 400 nL/min for 48 min at 5–80% solvent B, followed by running a linear gradient to 80% for 7 min, maintained at 80% B for 3 min, and finally returned to 5% in 7 min.

The peptides were subjected to nano-electrospray ionization followed by tandem mass spectrometry (MS/MS) in a Q EXACTIVE (Thermo Fisher Scientific, San Jose, CA, USA) coupled to the HPLC. Intact peptides were detected in the Orbitrap at a resolution of 70,000 and a mass range of 350–2000 m/z. Peptides were selected for MS/MS using high-energy collision dissociation (HCD), and ion fragments were detected in the Orbitrap at a resolution of 17,500. The electrospray voltage applied was 1.8 kV. MS/MS analysis was required for the 15 most abundant precursor ions, which were above a threshold ion count of 20,000 in the MS survey scan, including a following dynamic exclusion duration of 15 s.

### iTRAQ data analysis

The raw data files acquired from the mass spectrometers were converted into MGF files using 5600 MS Converter. Protein identification and quantification were performed using the Mascot Server (http://www.matrixscience.com/search_form_select.html) against the Uniprot_2015_human database (Matrix Science, London, UK; version 2.3.0) and Proteome Discoverer 1.3 (Thermo Fisher Scientific Inc.). To reduce the probability of false peptide identification, only peptides with significance scores at the 95% confidence interval as determined by a Mascot probability analysis were included. The quantitative protein ratios were weighted and normalized by the median ratio in Mascot. Statistical significance analyses were evaluated using two-way ANOVA. The proteins were considered to be differentially expressed if the ratio of mean fold change > 1.2 (or < 0.83) with an Exp pr > 0.05 and a Group pr < 0.05 (Exp pr, three-experiment *p* value; Group pr, group *p* value; fold change = experiment + group + error).

The Gene Ontology (GO) annotation of the identified proteins was performed via the online GO program (http://geneontology.org/). The biological functions, networks, and signaling pathways of the differentially expressed proteins (DEPs) were analyzed with Ingenuity Pathways Analysis (IPA) software (version 7.5, http://www.ingenuity.com) (Additional files 8, 9 and 10).

### RNA extraction and quantitative real-time PCR

Total RNA from the uninfected or infected cells was extracted with RNAiso Plus reagent according to the manufacturer’s instructions (TakaRa, Japan). Any genomic DNA contamination was eliminated by DNase I treatment, and the RNA was reverse-transcribed into cDNA using the PrimeScript™ RT reagent kit with gDNA Eraser, following the manufacturer’s instructions (Takara, Japan). Quantitative real-time PCR was performed in triplicate using the Power SYBR Green PCR Master Mix (Applied BioSystems, Foster City, CA, USA). The PCR primers for these experiments are listed in Table 1. The expression levels of the target genes were normalized to GAPDH by the 2^−ΔΔCT^ method.

**Table 1:** Primers used for real-time PCR in this study

Primers	Nucleotide sequence(5′-3′)	Gene symbol(s)
P1	ACGAATCTCCGACCACT	IL-1β
P2	CCATGGCCACAACAACTGAC	
P3	CTCAGCCTCTTCTCCTTC	TNF-α
P4	GGGTTTGCTACAACATGG	
P5	CCACTCACCTCTTCAGAA	IL-6
P6	GGCAAGTCTCCTCATTGA	
P7	GACATACTCCAAACCTTTCC	IL-8
P8	ATTCTCAGCCCTCTTCAAA	
P9	TGCCTCCTGCACCACCAACT	GAPDH
P10	CGCCTGCTTCACCACCTTC	

### Western blotting

Uninfected and infected hBMECs were collected and lysed in RIPA buffer supplemented with a protease inhibitor cocktail (Sigma-Aldrich, St. Louis, MO, USA) and then sonicated and centrifuged at 10,000×*g* for 10 min at 4 °C. The soluble protein concentration in the supernatants was measured using the BCA protein assay kit (Beyotime, China). Aliquots from each sample were separated by 12% SDS-PAGE, and then transferred to polyvinylidene difluoride membranes (Bio-Rad, CA, USA). The blots were blocked with 5% BSA in Tris-buffered saline with Tween 20 at room temperature for 1 h and then incubated overnight at 4 °C with primary antibodies against GAPDH, DMD, MIF, HIST1H1C, TBPL1 or LGMN. The blots were subsequently washed and incubated with horseradish peroxidase-conjugated anti-rabbit or anti-mouse IgG at 37 °C for 1 h, and visualized with ECL reagents (Bio-Rad, USA). The blots were densitometrically quantified and analyzed with Image Lab software (Bio-Rad).

### Immunofluorescence microscopy

Uninfected and infected hBMECs were fixed with 4% paraformaldehyde and permeabilized with 0.2% Triton X-100. After 2 h of blocking in PBS buffer with 5% BSA, the cells were incubated with the primary antibody (1:100) overnight at 4 °C, washed thrice with PBS, and then incubated with fluorescently labeled anti-mouse or anti-rabbit IgG (1500) for 1 h. Nuclei were stained with DAPI (0.5 μg/mL) for 30 min. Finally, the cells were mounted and then visualized with fluorescence microscopy.

### Electric cell substrate impedance sensing (ECIS)

To explore the influence of recombinant MIF on the permeability of the BBB, hBMECs were seeded at 7 × 10^4^ cells on collagen-coated, gold-plated electrodes in 96-well chamber slides (96W1E+) linked to ECIS Zθ equipment (Applied BioPhysics, Troy, NY, USA) and continuously cultured until confluence, and the trans-endothelial electric resistance (TEER) was monitored to reflect the formation of the barrier [28]. After stable maximal TEER was reached, the recombinant human MIF protein was added into the cells at multiple dosages (10, 100, and 200 ng/mL), and the possible TEER alteration of the monolayer cells was automatically recorded by the ECIS system.

### Statistical analysis

Data were expressed as the mean ± standard deviation (mean ± SD) from three replicates. Statistical significance of the differences between each group was analyzed by a one-way analysis of variance (ANOVA) or two-way ANOVA embedded in GraphPad Prism, version 6.0 (GraphPad Software Inc., La Jolla, CA, USA). *P* < 0.05 (*) was considered statistically significant, and *p* < 0.01 (**), as well as *p* < 0.001 (***) were all considered extremely significant.

## Results

### Differential protein profiling of hBMECs in response to *E. coli* infection

**Fig. 1:**
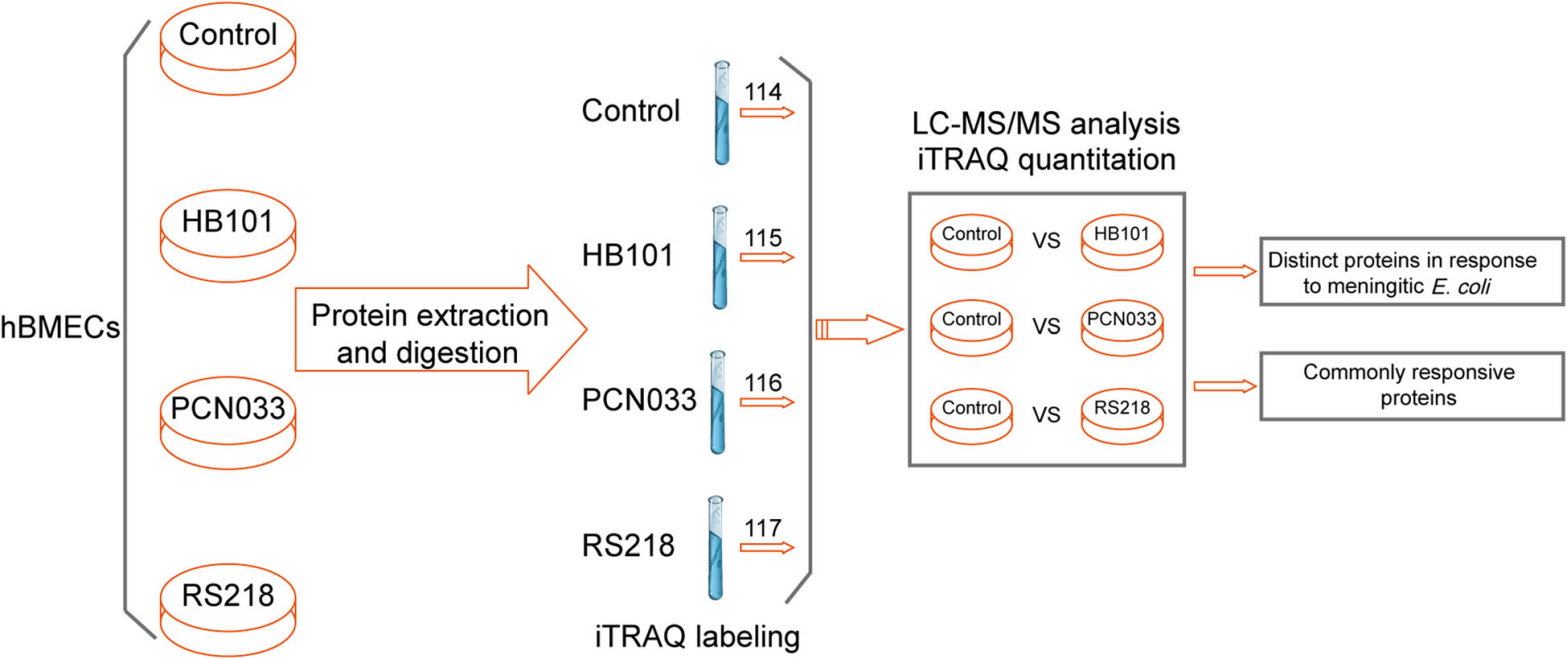
The general work flow for the proteomics analysis in this study

**Fig. 2:**
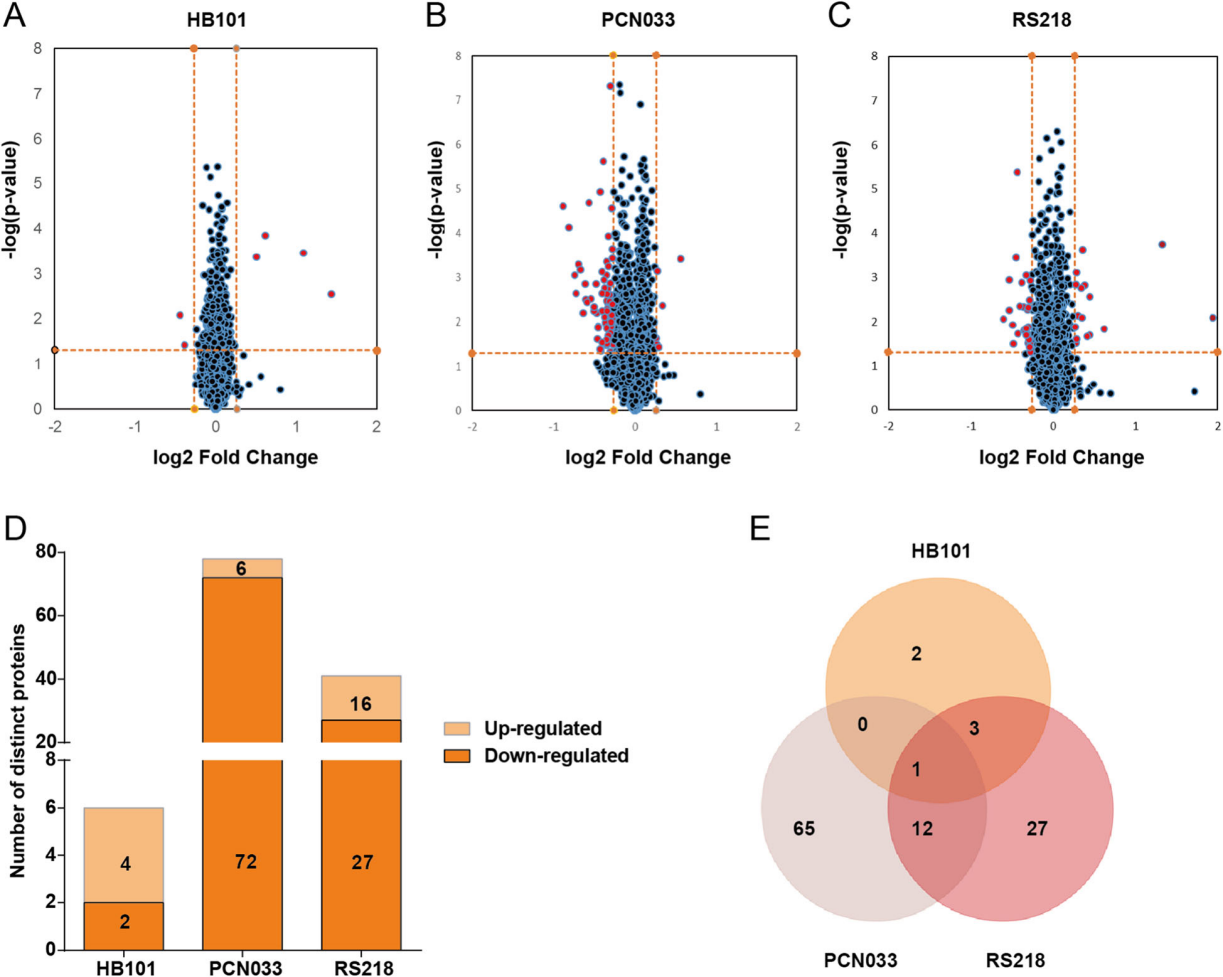
An overview of the DEPs in hBMECs in response to infection with meningitic *E. coli* strains PCN033 and RS218, and non-meningitic *E. coli* HB101. **a**–**c** The volcano plots show the cellular protein profiles in hBMECs after challenge with the three strains. **d** The number of significantly up- or downregulated proteins in the three infection groups. **e** Venn diagram showing the overlapping or distinct cellular proteins among the three groups

The protein extracts prepared from the hBMECs with or without meningitic *E. coli* challenge were subjected to the iTRAQ proteomics analysis. The whole work flow was shown in Fig. 1. Approximately 3000 different proteins were identified and quantified by iTRAQ-coupled LC-MS/MS analysis of the hBMECs infected with *E. coli* HB101, PCN033, or RS218 strains (Additional file 1: Table S1, Additional file 2: Table S2, Additional file 3: Table S3). As shown in Fig. 2a–d, four proteins were identified as being significantly upregulated and two were significantly downregulated upon HB101 infection, six were significantly upregulated, and 72 were significantly downregulated upon PCN033 infection, while 16 significantly upregulated and 27 significantly downregulated proteins were identified in cells challenged with RS218. The details of these differentially expressed proteins (DEPs) are listed in Tables 2, 3, and 4. The meningitic *E. coli* PCN033 group displayed 65 unique proteins, while the RS218 group displayed 27 unique proteins. They both shared 13 DEPs with 12 of them being distinct proteins in the hBMECs in response to meningitic strains PCN033 and RS218 (Fig. 2e, Table 5). Only one protein, EXOSC4, was shared by the three groups, and it showed a 0.74-, 0.759-, and 0.8-fold decrease in HB101, PCN033 and RS218 groups, respectively (Fig. 2e, Table 5). In contrast, infection with the non-meningitic HB101 strain induced only two unique, differentially altered proteins. Four proteins were shared between HB101 and RS218 groups, and the three of them altered in response to HB101 and RS218 were specific host proteins in both of these human isolates (Fig. 2e).

**Table 2:** Significantly changed proteins in HB101-infected hBMECs

Accession	Description	MW [kDa]	Fold change	*P* value^a^
P02656	Apolipoprotein C-III	10.8	1.544	0.000144***
Q07020	60S ribosomal protein L18	21.6	1.422	0.000429***
Q96HP4	Oxidoreductase NAD-binding domain-containing protein 1	34.8	2.715	0.002873**
Q9NPD3	Exosome complex component RRP41	26.4	0.737	0.008415**
Q9Y2Q5	Ragulator complex protein LAMTOR2	13.5	0.769	0.038574*
O14556	Glyceraldehyde-3-phosphate dehydrogenase, testis-specific	44.5	2.134	0.000347***

^a^*P* < 0.05 (*) was considered significant, and *P* < 0.01 (**), as well as < 0.001 (***) were all considered extremely significant

**Table 3:** Significantly changed proteins in PCN033-infected hBMECs

Accession	Description	MW [kDa]	Fold change	*P* value^a^
A6ZKI3	Protein FAM127A	13.2	0.756	0.028947*
O00625	Pirin	32.1	1.228	0.037445*
O43633	Charged multivesicular body protein 2a	25.1	0.758	0.002376**
O43752	Syntaxin-6	29.2	0.79	0.025619*
O60524	Nuclear export mediator factor NEMF	122.9	0.83	0.004194**
O75190	DnaJ homolog subfamily B member 6	36.1	0.799	0.020038*
O75251	NADH dehydrogenase [ubiquinone] iron-sulfur protein 7, mitochondrial	23.5	0.711	0.004822**
O75817	Ribonuclease P protein subunit p20	15.6	0.815	0.017814*
O95229	ZW10 interactor	31.3	0.668	0.003727**
P04004	Vitronectin	54.3	0.808	0.003548**
P07305	Histone H1.0	20.9	0.776	0.008176**
P11532	Dystrophin	426.5	0.691	0.003088**
P14174	Macrophage migration inhibitory factor	12.5	1.486	0.000377***
P16401	Histone H1.5	22.6	0.631	0.000673***
P16402	Histone H1.3	22.3	0.6	0.000896***
P16403	Histone H1.2	21.4	0.572	7.57E−05***
P35251	Replication factor C subunit 1	128.2	0.786	0.013936*
P35527	Keratin, type I cytoskeletal 9	62	0.72	0.001431**
P39060	Collagen alpha-1(XVIII) chain	178.1	0.792	0.031694*
P46013	Antigen KI-67	358.5	0.793	0.005934**
P48651	Phosphatidylserine synthase 1	55.5	0.83	0.000234***
P49585	Choline-phosphate cytidylyltransferase A	41.7	0.608	0.002306**
P50914	60S ribosomal protein L14	23.4	0.71	0.005946**
P52756	RNA-binding protein 5	92.1	0.758	0.005815**
P56377	AP-1 complex subunit sigma-2	18.6	0.765	2.4E−06***
P61966	AP-1 complex subunit sigma-1A	18.7	0.814	4.81E−08***
P62277	40S ribosomal protein S13	17.2	0.792	0.002441**
P62380	TATA box-binding protein-like protein 1	20.9	0.621	0.000503***
Q13625	Apoptosis-stimulating of p53 protein 2	125.5	0.724	0.006481**
Q14241	Transcription elongation factor B polypeptide 3	89.9	0.647	0.006556**
Q14686	Nuclear receptor coactivator 6	219	0.792	0.005234**
Q15388	Mitochondrial import receptor subunit TOM20 homolog	16.3	0.823	0.007395**
Q15629	Translocating chain-associated membrane protein 1	43	0.809	0.023084*
Q17RN3	Protein FAM98C	37.3	0.821	0.010317*
Q4V339	COBW domain-containing protein 6	43.9	0.747	1.2E−05***
Q567U6	Coiled-coil domain-containing protein 93	73.2	0.814	0.001351**
Q5SSJ5	Heterochromatin protein 1-binding protein 3	61.2	0.828	0.000364***
Q6N069	*N*-alpha-acetyltransferase 16, NatA auxiliary subunit	101.4	0.775	0.001734**
Q709C8	Vacuolar protein sorting-associated protein 13C	422.1	0.799	0.000576***
Q7Z422	SUZ domain-containing protein 1	17	0.808	0.002542**
Q8IXJ9	Putative Polycomb group protein ASXL1	165.3	0.807	0.007037**
Q8N2K0	Monoacylglycerol lipase ABHD12	45.1	0.786	0.0034**
Q8N884	Cyclic GMP-AMP synthase	58.8	0.82	0.013058*
Q8NC44	Protein FAM134A	57.8	0.78	0.010814*
Q8NC60	Nitric oxide-associated protein 1	78.4	0.81	0.013637*
Q8NEY1	Neuron navigator 1	202.3	0.797	0.020924*
Q8TEM1	Nuclear pore membrane glycoprotein 210	205	0.833	0.032212*
Q8WUP2	Filamin-binding LIM protein 1	40.6	0.809	0.002633**
Q8WVV9	Heterogeneous nuclear ribonucleoprotein L-like	60	0.804	0.013638*
Q8WXA3	RUN and FYVE domain-containing protein 2	75	0.744	0.041934*
Q92604	Acyl-CoA:lysophosphatidylglycerol acyltransferase 1	43.1	0.789	0.033787 *
Q96A57	Transmembrane protein 230	13.2	0.786	0.000449***
Q96LB3	Intraflagellar transport protein 74 homolog	69.2	0.543	2.53E−05***
Q96RU3	Formin-binding protein 1	71.3	0.679	2.07E−05***
Q96T37	Putative RNA-binding protein 15	107.1	0.728	0.024086*
Q9GZP8	Immortalization upregulated protein	10.9	1.207	0.032624*
Q9H074	Polyadenylate-binding protein-interacting protein 1	53.5	1.266	0.004395**
Q9H5N1	Rab GTPase-binding effector protein 2	63.5	0.77	0.001156**
Q9H5X1	MIP18 family protein FAM96A	18.3	0.8	0.000118***
Q9HB40	Retinoid-inducible serine carboxypeptidase	50.8	1.215	0.000733***
Q9HC52	Chromobox protein homolog 8	43.4	1.201	0.023152*
Q9NPD3	Exosome complex component RRP41	26.4	0.759	0.000746***
Q9NRY4	Rho GTPase-activating protein 35	170.4	0.792	0.011172*
Q9NS87	Kinesin-like protein KIF15	160.1	0.785	0.010039*
Q9NSP4	Centromere protein M	19.7	0.802	0.021316*
Q9NTI5	Sister chromatid cohesion protein PDS5 homolog B	164.6	0.826	0.003399**
Q9NWU5	39S ribosomal protein L22, mitochondrial	23.6	0.812	0.016677*
Q9NZQ3	NCK-interacting protein with SH3 domain	78.9	0.661	0.00317**
Q9P0V3	SH3 domain-binding protein 4	107.4	0.797	0.001833**
Q9UBL6	Copine-7	70.2	0.823	2.73E−05***
Q9UJW0	Dynactin subunit 4	52.3	0.823	0.012604*
Q9UNP9	Peptidyl-prolyl cis-trans isomerase E	33.4	0.75	0.044207*
Q9Y2R0	Cytochrome c oxidase assembly protein 3 homolog, mitochondrial	11.7	0.792	0.003694**
Q9Y5Y2	Cytosolic Fe-S cluster assembly factor NUBP2	28.8	0.787	0.000891***
Q9Y6I9	Testis-expressed sequence 264 protein	34.2	0.814	0.047637*
Q9Y3Y2	Chromatin target of PRMT1 protein	26.4	0.828	0.008622**
Q9Y4R8	Telomere length regulation protein TEL2 homolog	91.7	0.735	0.013443*
P10412	Histone H1.4	21.9	0.655	0.001429**

^a^*P* < 0.05 (*) was considered significant, and *P* < 0.01 (**), as well as < 0.001 (***), were all considered extremely significant

**Table 4:** Significantly changed proteins in RS218-infected hBMECs

Accession	Description	MW [kDa]	Fold change	*P* value^a^
O00592	Podocalyxin	58.6	1.214	0.001481**
O14556	Glyceraldehyde-3-phosphate dehydrogenase, testis-specific	44.5	2.514	0.000183***
O43598	2′-Deoxynucleoside 5′-phosphate *N*-hydrolase 1	19.1	0.8	0.020803*
O76024	Wolframin	100.2	0.732	0.000347***
O76095	Protein JTB	16.3	0.815	0.026287*
O95989	Diphosphoinositol polyphosphate phosphohydrolase 1	19.5	0.821	0.003332**
P05067	Amyloid beta A4 protein	86.9	0.813	0.004913**
P10412	Histone H1.4	21.9	1.271	0.001736**
P11532	Dystrophin	426.5	0.799	0.014535*
P14174	Macrophage migration inhibitory factor	12.5	1.276	0.008267**
P16401	Histone H1.5	22.6	1.221	0.025445*
P16402	Histone H1.3	22.3	1.306	0.001514**
P16403	Histone H1.2	21.4	1.332	0.021727*
P30154	Serine/threonine-protein phosphatase 2A 65 kDa regulatory subunit A beta isoform	66.2	0.809	0.005474**
P35527	Keratin, type I cytoskeletal 9	62	0.822	0.038701*
P42167	Lamina-associated polypeptide 2, isoforms beta/gamma	50.6	0.826	0.0494*
P46781	40S ribosomal protein S9	22.6	1.207	0.013518*
P50402	Emerin	29	0.8	0.000916***
P52756	RNA-binding protein 5	92.1	0.74	4.23E−06***
P55789	FAD-linked sulfhydryl oxidase ALR	23.4	1.537	0.014932*
P61313	60S ribosomal protein L15	24.1	1.286	0.000236***
P62380	TATA box-binding protein-like protein 1	20.9	0.66	0.008696**
Q07020	60S ribosomal protein L18	21.6	1.367	0.002799**
Q4V339	COBW domain-containing protein 6	43.9	0.756	0.00457**
Q8N4H5	Mitochondrial import receptor subunit TOM5 homolog	6	1.223	0.000783***
Q8ND56	Protein LSM14 homolog A	50.5	0.793	0.017061*
Q96BZ8	Leukocyte receptor cluster member 1	30.5	0.693	0.005542**
Q96HP4	Oxidoreductase NAD-binding domain-containing protein 1	34.8	3.845	0.008111**
Q96KR1	Zinc finger RNA-binding protein	116.9	0.811	0.01368*
Q96LB3	Intraflagellar transport protein 74 homolog	69.2	0.71	0.012006*
Q96P47	Arf-GAP with GTPase, ANK repeat and PH domain-containing protein 3	95	0.783	0.004713**
Q99538	Legumain	49.4	0.692	0.001173**
Q9BTA9	WW domain-containing adapter protein with coiled-coil	70.7	0.743	0.01886*
Q9BZF9	Uveal autoantigen with coiled-coil domains and ankyrin repeats	162.4	0.824	0.031539*
Q9H7B2	Ribosome production factor 2 homolog	35.6	1.367	0.020862*
Q9HCD5	Nuclear receptor coactivator 5	65.5	0.771	0.001324**
Q9NPD3	Exosome complex component RRP41	26.4	0.784	0.015931*
Q9NZR1	Tropomodulin-2	39.6	1.216	0.030742*
Q9UI10	Translation initiation factor eIF-2B subunit delta	57.5	0.828	0.001214**
Q9UIC8	Leucine carboxyl methyltransferase 1	38.4	0.811	0.0312*
Q9UK41	Vacuolar protein sorting-associated protein 28 homolog	25.4	0.715	0.032545*
Q9Y4R8	Telomere length regulation protein TEL2 homolog	91.7	0.807	0.019858*
Q9Y5V3	Melanoma-associated antigen D1	86.1	1.238	0.007536**

^a^*P* < 0.05 (*) was considered significant, and *P* < 0.01 (**), as well as < 0.001 (***), were all considered extremely significant

**Table 5:** The distinct differential proteins in hBMECs in response to meningitic *E. coli* strains PCN033 and RS218

ID	Name	Protein	Fold Change		
			RS218	PCN033	HB101
Q9NPD3	EXOSC4	Exosome complex component RRP41	0.8	0.759	0.74
Q96LB3	IFT74	Intraflagellar transport protein 74 homolog	0.7	0.543	/
P11532	DMD	Dystrophin	0.8	0.691	/
P52756	RBM5	RNA-binding protein 5	0.7	0.758	/
Q4V339	CBWD6	COBW domain-containing protein 6	0.8	0.747	/
Q9Y4R8	TELO2	Telomere length regulation protein TEL2 homolog	0.8	0.735	/
P35527	KRT9	Keratin, type I cytoskeletal 9	0.8	0.72	/
P62380	TBOL1	TATA box-binding protein-like protein 1	0.7	0.621	/
P16403	HIST1H1C	Histone H1.2	1.3	0.572	/
P16402	HIST1H1D	Histone H1.3	1.3	0.6	/
P10412	HIST1H1E	Histone H1.4	1.3	0.655	/
P16401	HIST1H1B	Histone H1.5	1.2	0.631	/
P14174	MIF	Macrophage migration inhibitory factor	1.3	1.486	/

### Western blot verification of the DEPs

**Fig. 3:**
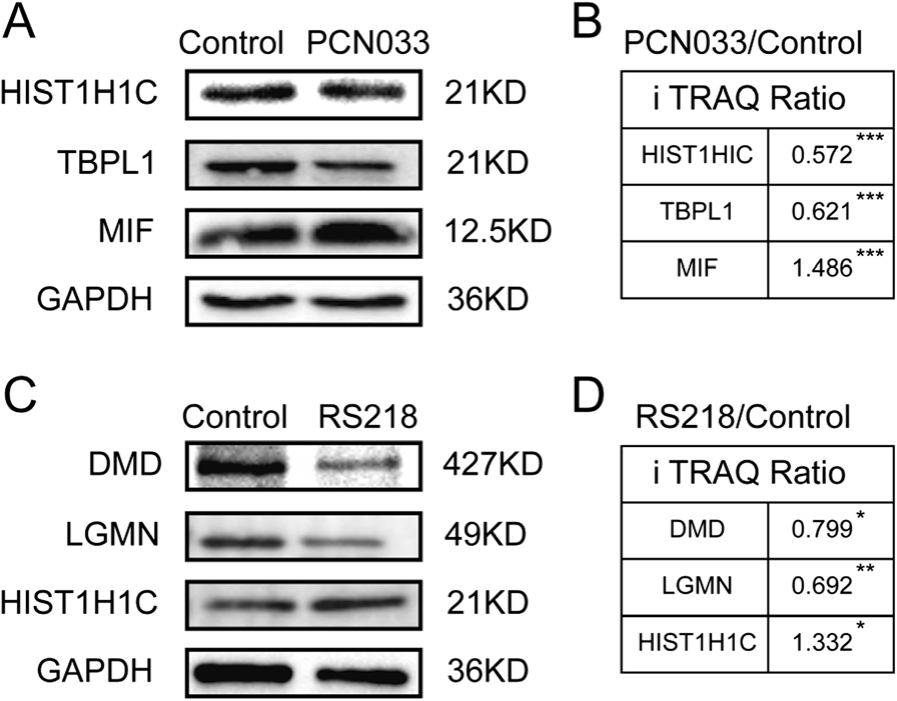
DEPs validation. **a** Immunoblotting analysis of the DEPs (HIST1H1C, TBPL1, and MIF) in the hBMECs with or without PCN033 infection. **b** iTRAQ ratios of the DEPs in hBMECs with PCN033 infection. **c** Immunoblotting analysis of the DEPs (DMD, LGMN, and HIST1H1C) in hBMECs with or without RS218 infection. **d** iTRAQ ratios of the DEPs in hBMECs with RS218 infection. *(*P* < 0.05) was considered statistically significant; ** (*p* < 0.01) and *** (*p* < 0.001) were extremely significant

We next used western blotting to further test the DEPs identified by iTRAQ. We selected several proteins from the iTRAQ results from both PCN033 and RS218 groups. The test proteins were HIST1H1C, TBPL1, and MIF for the PCN033 group (Fig. 3a), and DMD, LGMN, and HIST1H1C for the RS218 group (Fig. 3c). The western blot and densitometry analyses produced the similar expression alteration to those of the iTRAQ results following either PCN033 or RS218 infection (Fig. 3b, d).

### Bioinformatic analysis of the DEPs in hBMECs

**Fig. 4:**
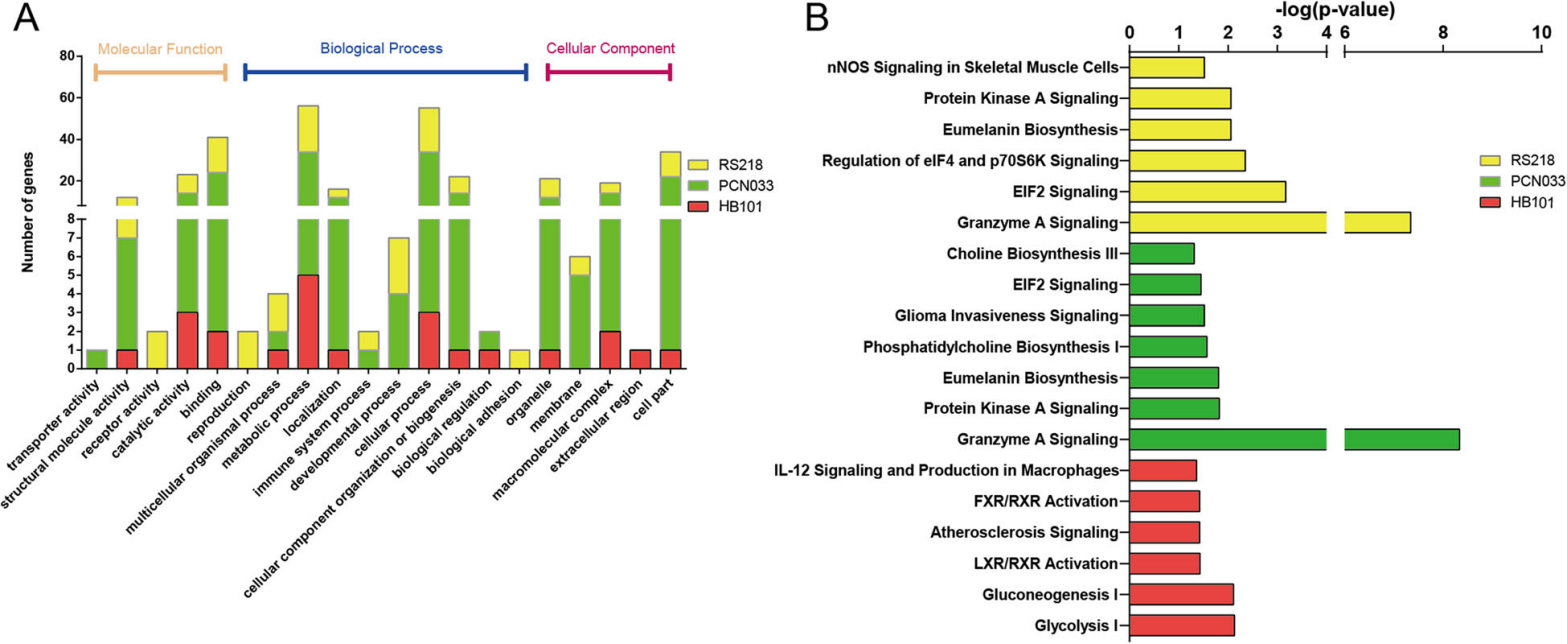
GO annotation and pathway enrichment comparison of DEPs upon meningitic or non-meningitic *E. coli* infection. **a** GO annotation characterization of the molecular functions, biological processes, and cellular components based on the DEPs. **b** Pathway enrichment of cellular DEPs in response to infection with HB101, PCN033, and RS218 strains

We next investigated and characterized the DEPs by searching the GO and UniProt databases. The DEPs were assigned to the categories of different “biological processes,” “cellular components,” and “molecular functions.” Within the biological processes class, the DEPs from the three groups (RS218, PCN033, and HB101) were mainly divided into metabolic processes, localization, cellular process, and cellular component organization or biogenesis. The immune system process and developmental process classes were found in both RS218 and PCN033 infection groups, but not in the HB101 group. Within the cellular component class, the DEPs were mainly divided into organelle, macromolecular complex, and cell parts, and the membrane-associated ones were only identified in the meningitic strains RS218 and PCN033, not in HB101. As for molecular function, the DEPs were mainly associated with structural molecule activity, catalytic activity, and binding (Fig. 4a, Additional file 4: Table S4).

We next performed canonical pathway prediction through IPA on the DEPs. The top ranked canonical pathways in each group are shown in Fig. 4b. We found that protein kinase A signaling, eumelanin biosynthesis, EIF2 signaling, and granzyme A signaling were simultaneously enriched in both RS218 and PCN033 infection groups, but not in the HB101 group (Fig. 4b). Noticeably, granzyme A signaling was much more significantly enriched in the DEPs from both meningitic groups, suggesting a potential role for granzyme A in meningitic *E. coli* invasion of the BBB. Additionally, phosphatidylcholine biosynthesis I, choline biosynthesis III, and glioma invasiveness signaling were only enriched in the PCN033 group, while neuronal NOS signaling and regulation of eIF4 and p70S6K signaling were only identified in the RS218 group, which exhibited distinct signaling pathways that might have strain specificity (Fig. 4b).

**Fig. 5:**
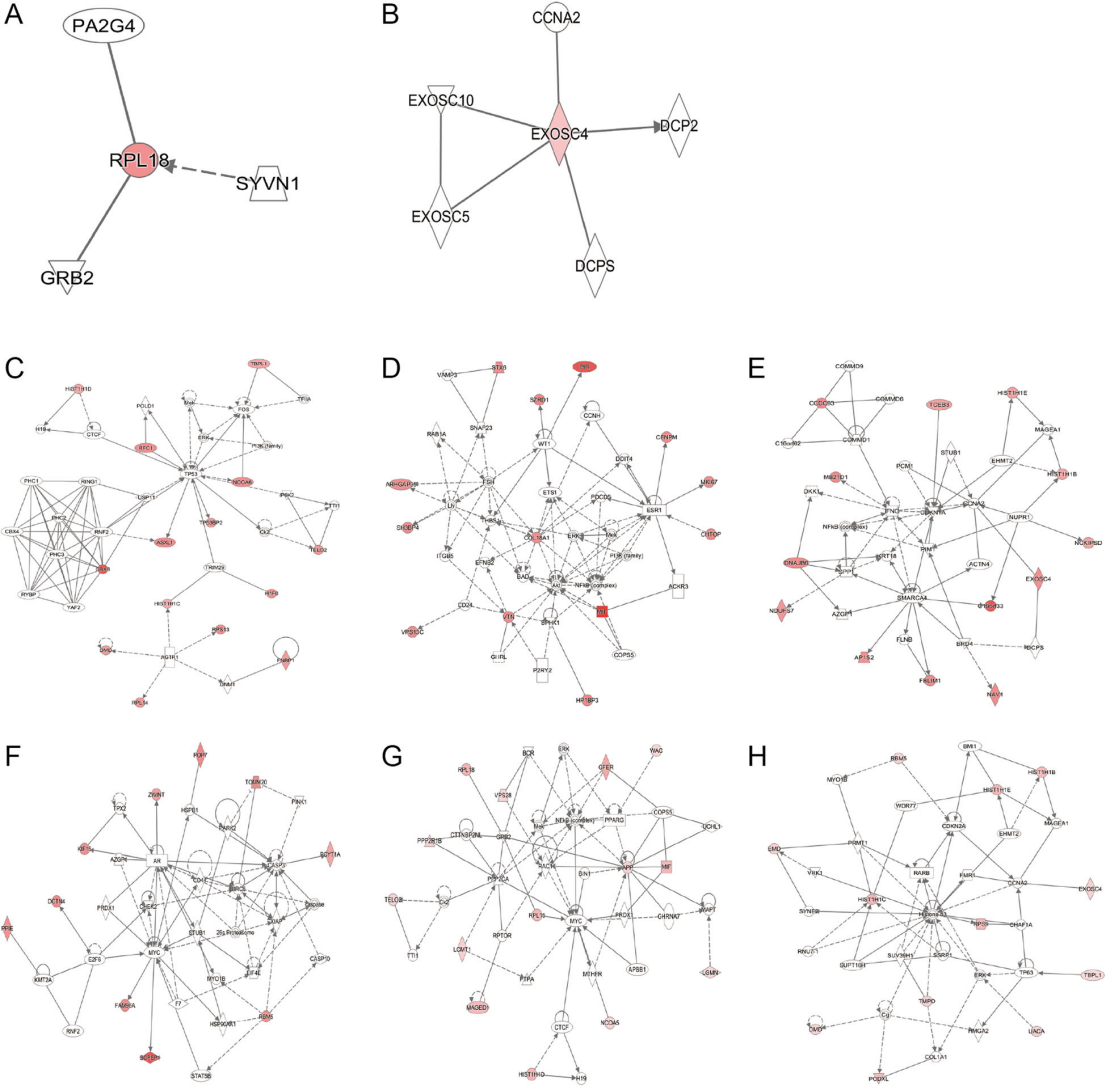
Network analysis of significantly altered proteins in hBMECs upon *E. coli* infection. For the HB101 infection, two networks were constructed: **a** cellular assembly and organization, gastrointestinal disease, hepatic system disease; **b** RNA damage and repair, connective tissue disorders, developmental disorder. For the PCN033 infection, four networks were constructed: **c** lymphoid tissue structure and development, organ morphology, organismal development; **d** cellular movement, cancer, organismal injury and abnormalities; **e** inflammatory disease, inflammatory response, organismal injury and abnormalities; **f** cell death and survival, cellular development, cellular growth and proliferation. For the RS218 infection two networks were constructed: **g** neurological disease, organismal injury and abnormalities, cell cycle; **h** gene expression, cellular assembly and organization, DNA replication, recombination, and repair. The red nodes indicate significantly altered protein expression, and the white ones are those known to be involved in the networks, but not identified in this study. Arrows indicate the interrelationship between two molecules. Solid lines indicate direct interactions and dashed lines indicate indirect interactions

The IPA tool was used to further analyze the potential networks based on the DEPs from the *E. coli* infections. Two networks were drawn for these differential cellular proteins in response to HB101 infection (Fig. 5a, b, Additional file 5: Table S5). In addition, four networks were generated based on the DEPs from the PCN033 infection (Fig. 5c–f, Additional file 6: Table S6), while two networks were generated from the DEPs upon RS218 infection (Fig. 5g, h, Additional file 7: Table S7). It should be noted that the NF-κB complex, as well as ERK, were included in the networks of both PCN033 and RS218 groups, while they were not observed in the cells in response to the non-meningitic HB101 strain, suggesting that these two essential signaling molecules exert regulatory effects during meningitic *E. coli* penetration of the BBB.

### MIF contributes to meningitic *E. coli*-induced cytokine production and tight junction disruption

**Fig. 6:**
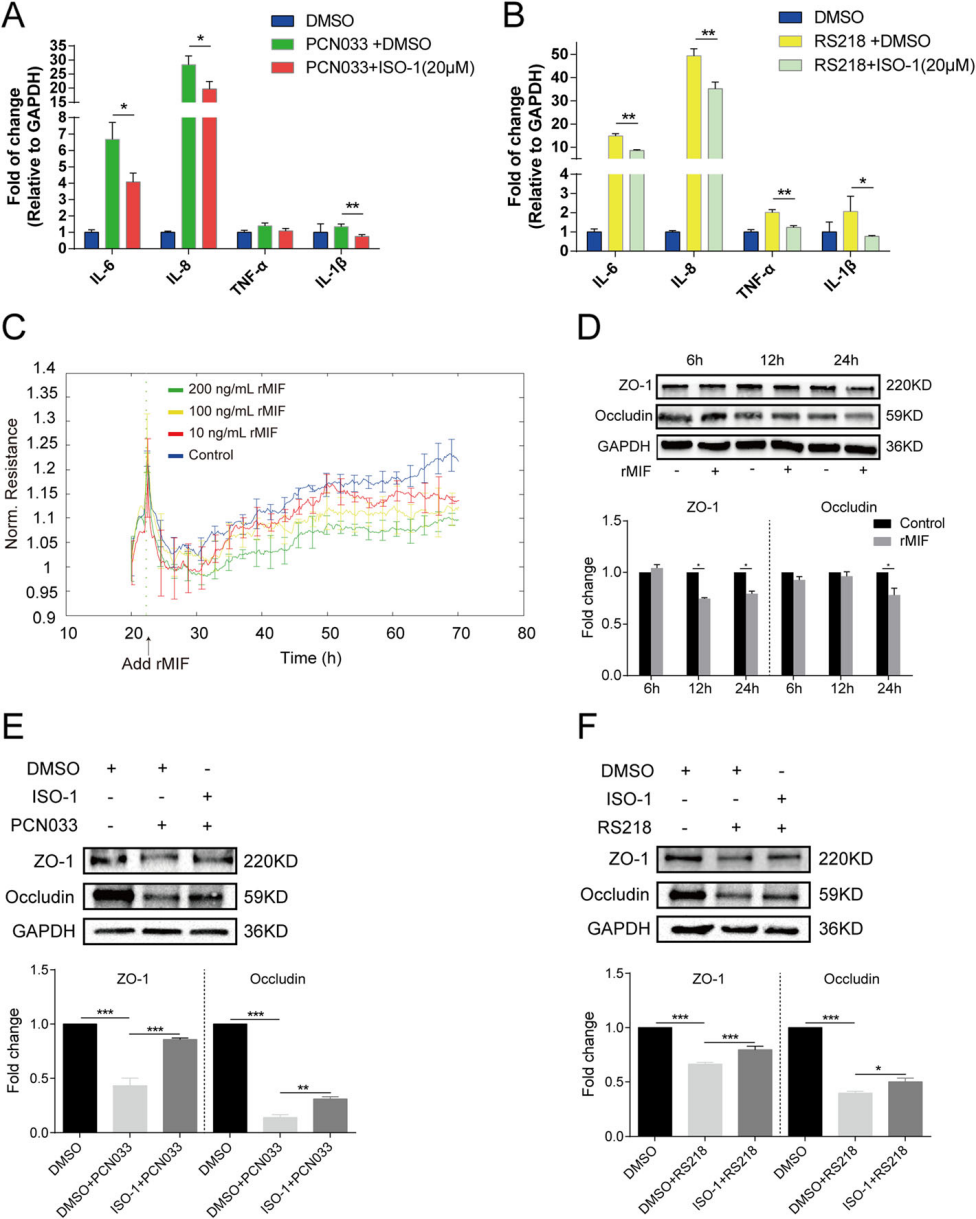
MIF facilitated the bacteria-induced inflammatory response and tight junction damage in hBMECs. **a**, **b** Real-time PCR determination of the expression of cytokines in response to the treatments. The MIF inhibitor ISO-1 (20 μM) significantly attenuated the PCN033- or RS218-induced production of proinflammatory cytokines. **c** ECIS assay showed a dose-dependent decrease of the hBMECs resistance in response to recombinant MIF protein. **d** Recombinant MIF protein (200 ng/mL) decreased the expression of tight junction proteins ZO-1 and occludin in hBMECs along with time. The densitometry was performed to quantitatively analyze the Western bands. **e**, **f** Western blotting and densitometry analysis showed that ISO-1 treatment partially recovered PCN033- or RS218-mediated downregulation of the tight junction proteins ZO-1 and occludin. Data were expressed as the mean ± standard deviation (mean ± SD) from three replicates or analyses (*n* = 3). *P* < 0.05 (*) was considered statistically significant; *p* < 0.01 (**) and *p* < 0.001 (***) were extremely significant

Based on the aforementioned network analysis, we noticed the presence of MIF in the meningitic PCN033 and RS218 strain groups, suggesting potential roles for it in meningitic *E. coli* invasion of the BBB. Here, by pretreating the hBMECs with 20 μM ISO-1 (a MIF inhibitor), we found that the multiple cytokines [e.g. interleukin (IL)-6, IL-8, tumor necrosis factor (TNF)-α, IL-1β] significantly induced by meningitic *E. coli* PCN033 or RS218 infection had decreased levels (Fig. 6a, b). Moreover, the ECIS system was applied to evaluate the potential effects of recombinant MIF protein on the barrier function of hBMECs. The results showed that recombinant MIF obviously decreased the resistance formed by the cells in a dose-dependent manner (Fig. 6c). We also observed that treatment with recombinant MIF (200 ng/ml) for 12 and 24 h led to decreased expression of tight junction proteins like ZO-1 and occludin (Fig. 6d); moreover, use of the MIF inhibitor ISO-1 could partially recover the PCN033 or RS218 infection-caused downregulation of tight junction proteins like ZO-1 and Occludin (Fig. 6e, f). Together, these observations support the conclusion that MIF contributes to the induction of proinflammatory cytokines and the decrease in tight junction proteins during meningitic *E. coli* invasion of the BBB.

### Meningitic *E. coli* activation of NF-κB signaling mediates the production of cytokines

**Fig. 7:**
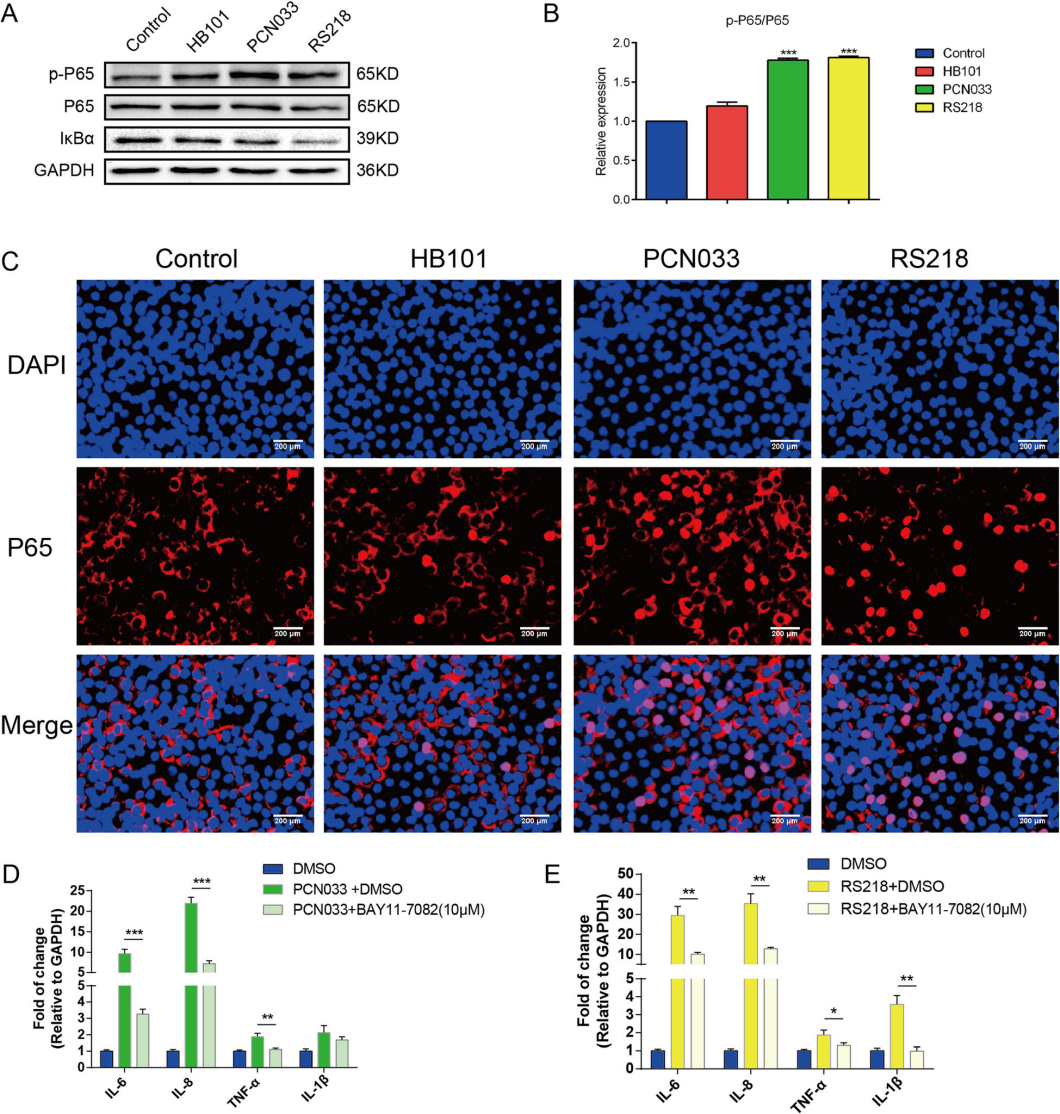
NF-κB signaling is activated in response to meningitic PCN033 or RS218 and mediates the inflammatory response. **a**, **b** p65 phosphorylation and IκBα degradation were significantly enhanced upon challenge with PCN033 and RS218, as shown by western blotting and densitometry. **c** Nuclear translocation of the p65 subunit was apparent in the hBMECs upon infection with PCN033 and RS218, but barely observed in response to infection with HB101. **d**, **e** Real-time PCR analysis showed that meningitic *E. coli* strains PCN033- and RS218-induced proinflammatory cytokines production was significantly decreased via NF-κB signaling inhibition with 10 μM of BAY11-7082. Data were expressed as the mean ± standard deviation (mean ± SD) from three replicates or analyses. *P* < 0.05 (*) was considered statistically significant; *p* < 0.01 (**) and *p* < 0.001 (***) were extremely significant

As mentioned above in the network analysis, involvement of the NF-κB complex was observed in cells following the challenge with meningitic *E. coli* strains PCN033 and RS218, but not with non-meningitic HB101. Therefore, we investigated NF-κB signaling activation in hBMECs in response to infection. Phosphorylation of the NF-κB p65 subunit increased significantly in response to PCN033 and RS218 infection, and this was much higher than that observed during the response to HB101 infection. Also, degradation of IκBα upon PCN033 or RS218 infection was much greater than that upon HB101 infection (Fig. 7a, b). Using immunofluorescence microscopy, we also observed p65 translocation to the nucleus upon PCN033 and RS218 infection (Fig. 7c), while this nuclear translocation was barely observed in response to HB101 infection (Fig. 7c). These results indicate that the NF-κB signaling pathway is activated during meningitic *E. coli* interaction with hBMECs. Moreover, by using the NF-κB inhibitor BAY11-7082, we observed that the meningitic *E. coli* PCN033- or RS218-induced cytokines production (including IL-6, IL-8, TNF-α, and IL-1β) was significantly decreased when compared with DMSO treatment (Fig. 7d, e). Together, these data firmly support our network analysis that the NF-κB signaling pathway is involved in both PCN033 and RS218 infection of hBMECs, and their activation of NF-κB signaling in hBMECs mediates the induction of proinflammatory cytokines.

### MAPK signaling pathways are involved in proinflammatory cytokine induction by meningitic *E. coli* strains

**Fig. 8:**
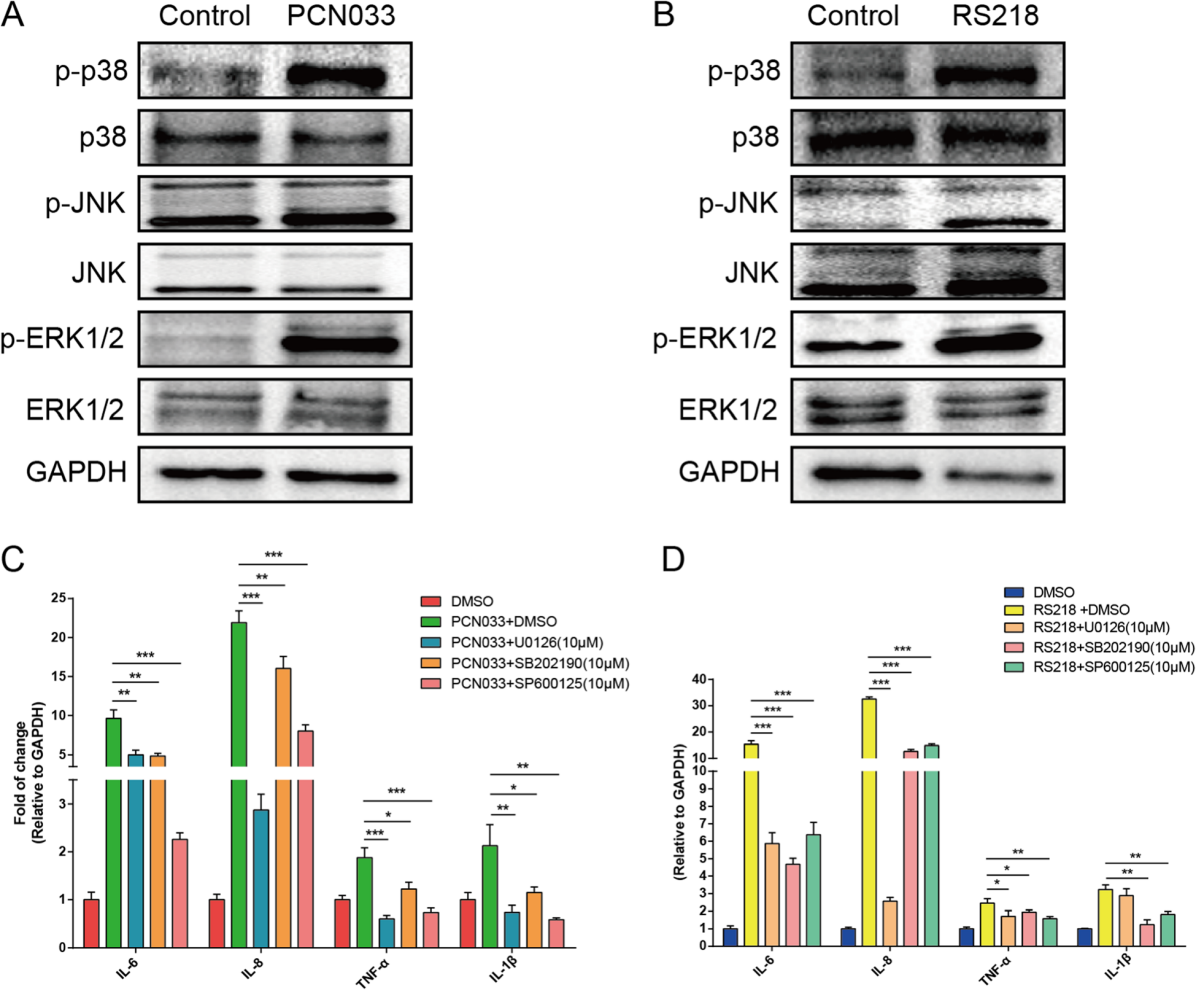
MAPK signaling, which is activated in hBMECs upon meningitic *E. coli* infection, contributes to the neuroinflammatory response. **a**, **b** Phosphorylation of p38, JNK, and ERK1/2 in hBMECs upon challenge with PCN033 and RS218 strains. **c**, **d** Blocking the three MAPK signaling pathways through specific inhibitors (U0126, a specific ERK1/2 inhibitor; SB202190, a p38 selective inhibitor; and SP600125, a JNK-specific inhibitor) significantly decreased the infection-induced neuroinflammatory response via real-time PCR analysis. *P* < 0.05 (*) was considered statistically significant; *p* < 0.01 (**) and *p* < 0.001 (***) were extremely significant

Because ERK was assumed to be involved in infections with PCN033 and RS218 based on our network prediction, we next investigated the activation of MAPK pathways in hBMECs in response to meningitic *E. coli*. The results showed that the phosphorylation of p38, JNK, and ERK1/2 significantly increased in response to meningitic strains PCN033 or RS218 (Fig. 8a, b), indicating the activation of all three MAPK pathways in hBMECs upon meningitic *E. coli* challenge. After demonstrating the significant induction of several proinflammatory cytokines above, we next investigated whether the MAPK pathways were involved in these cytokines production. Following pretreatment with U0126 (a specific ERK1/2 inhibitor), SB202190 (a selective inhibitor of p38), and SP600125 (a JNK-specific inhibitor), the proinflammatory cytokines (IL-6, IL-8, TNF-α, IL-1β) induced in hBMECs upon PCN033 or RS218 infection were significantly reduced (to different extents), compared with that in each DMSO control group (Fig. 8c). These results indicate that the MAPK signaling pathways, including MAPK-p38, MAPK-ERK1/2, and MAPK-JNK, were all activated and at least participated in meningitic *E. coli*-induced neuroinflammatory responses.

## Discussion

The iTRAQ-based proteomics, a powerful approach for obtaining comprehensive and quantitative protein expression profiling data, has been used widely to identify and characterize potential cellular targets. In current study, we used iTRAQ to explore the proteomic differences in hBMECs in response to meningitic or non-meningitic *E. coli* infections. The *E. coli* strains PCN033 and RS218 were selected for this study because they are representative meningitis-causing strains capable of penetrating the BBB as well as inducing severe neuroinflammation [5, 20], while the *E. coli* strain HB101 is avirulent and non-meningitic and was therefore used as the negative control.

Based on our data, 13 significantly differentiated proteins in total were found to be shared by PCN033 and RS218 (Fig. 1). They are TELO2, IFT74, CBWD6, EXOSC4, TBOL1, RBM5, KRT9, HIST1H1C, HIST1H1D, HIST1H1B, HIST1H1E, MIF, and DMD (Table 5). Among these, EXOSC4 was the only protein that was also significantly changed in response to non-meningitic *E. coli* HB101 (Fig. 2, Table 5). EXOSC4, a non-catalytic component of the RNA exosome machinery, has 3′-5′ exoribonuclease activity and participates in a multitude of cellular RNA processing and degradation events [29]. It was reported that EXOSC4 was a potential factor involved in the maintenance of genome stability, by eliminating the RNA processing by-products and non-coding “pervasive” transcripts thereby limiting or excluding their export to the cytoplasm, or by preventing translation of aberrant mRNAs [30–32]. In lung adenocarcinoma, EXOSC4 has been reported to be extremely highly expressed and closely associated with cancer cell proliferation and was, therefore, recognized as a new prognostic marker [30]. Similarly, in patients with liver cancer, the EXOSC4 gene was found to be highly expressed, and its knock-down commonly inhibited cancer cell growth and invasion [33]. Here, we found that EXOSC4 was commonly targeted by the meningitic and the non-meningitic *E. coli* strains, indicating that this cellular protein is a non-specific infection-related protein. Other than EXOSC4, the remaining 12 proteins were shared by the meningitic strains (PCN033 and RS218) alone, suggesting that these proteins might represent the potential targets hijacked by these meningitic *E. coli* strains.

Among these 12 meningitic *E. coli*-specific “cellular responders,” we firstly focused on MIF, which was the only one to exhibit common upregulation in response to both meningitic *E. coli* PCN033 and RS218 (Table 5). MIF is a proinflammatory cytokine, which has been highlighted as a key player in infection and septic shock [34, 35]. It is reported to be involved in the cytokine storm, which facilitates the uncontrolled release of cytokines into the circulation during pathogen infection or sepsis [36]. As previously evidenced in *E. coli*-induced meningitis, cytokines and chemokines potentially contribute to BBB damage [5]. The burst of proinflammatory cytokines during infection may lead directly to dysfunction of the endothelial barrier and an increase in vascular permeability in the brain, thus finally leading to severe CNS injury. Moreover, MIF may be secreted by a wide variety of cells upon stimulation, and once MIF binds to its receptors (e.g., CXCR2, CXCR4, and/or CD74 [37, 38]), several downstream signal molecules such as PI3K/Akt or MAPK/ERK become activated, thus mediating the inflammatory response [39, 40]. In the present study, the effects of MIF on meningitic *E. coli*-induced inflammation were also verified by the observation that the MIF inhibitor ISO-1 significantly decreased meningitic *E. coli* PCN033- or RS218-induced upregulation of IL-6, IL-8, IL-Iβ, and TNF-α (Fig. 5). Noticeably however, although the ISO-1 inhibitory effects were significant, there was still a significant induction of IL-6 and IL-8 in response to PCN033 and RS218 infection, suggesting that other “switches” for proinflammatory cytokine and chemokine generation commonly exist in response to infection. Except for its role in inflammation, we also observed the involvement of MIF in BBB damage, as evidenced by the fact that recombinant MIF was able to deconstruct the endothelial barrier by inducing a significant decrease in the junction-associated protein ZO-1 and occludin (Fig. 6). Furthermore, when MIF inhibitor ISO-1 was used, the PCN033- and/or RS218-induced downregulation of ZO-1 and occludin was largely restored (Fig. 6). Considering the potential roles of MIF in mediating the neuroinflammatory response as well as in inducing BBB disruption, it is possible that MIF may represent a novel and potential target for clinical prevention and therapy for *E. coli* meningitis.

Our IPA-based canonical pathways prediction suggested that protein kinase A signaling, eumelanin biosynthesis, EIF2 signaling, and granzyme A signaling were simultaneously enriched in hBMECs upon infection with RS218 and PCN033, but not with HB101. Among these processes, granzyme A signaling was much more significantly enriched. In the RS218 group, HIST1H1B, HIST1H1C, HIST1H1E, and HIST1H1D are included in granzyme A signaling, while in the PCN033 group, HIST1H1B, HIST1H1C, HIST1H1E, HIST1H1D, and H1F0 are involved (Additional file 6: Table S6). Granzyme A was identified as a cytotoxic T lymphocyte protease with multiple roles in infectious diseases. For example, several studies have shown that granzyme A is highly expressed in patients with tuberculosis and may represent a promising diagnostic marker distinct from IFN-γ to discriminate between patients with tuberculosis and other pulmonary diseases [41–43]. Granzyme A is also considered to participate in the host defense response in multiple ways, such as by generating superoxide and inactivating the oxidative defense enzymes that kill intracellular parasites [44], by unfavorably impairing host defenses during *Streptococcus pneumoniae* pneumonia [45], by performing as a proinflammatory protease that cleaves IL-1β intracellularly into bioactive IL-1β [46, 47], or by causing detachment of alveolar epithelial A549 cells accompanied by promotion of IL-8 release [48]. Here, in the present study, granzyme A signaling was significantly enriched by cellular differentiated proteins in response to both meningitic *E. coli* strains, but not in non-meningitic *E. coli* HB101. This result probably indicates that granzyme A could be a potential indicator of *E. coli* meningitis, but further supportive evidences are needed.

Based on the IPA functional network analysis, we also noticed that the NF-κB complex and MAPK/ERK signaling were involved in both PCN033 and RS218 infection of hBMECs, but barely in the HB101 group. The NF-κB complex comprises a family of closely related transcription factors with important roles in regulating the gene expression involved in inflammation and the immune response [49]. The NF-κB activation process is induced by the phosphorylation of serine residues in IkB proteins, which are subjected to ubiquitination and proteasome degradation and, subsequently, phosphorylation and nuclear translocation of the p65 subunit. Early studies have shown that NF-κB is activated in bacteria-induced CNS infections [50], and NF-κB inhibitors have been found to reduce neuroinflammation [51] as well as protect rat brains from inflammatory injury following transient focal cerebral ischemia [52] and pneumococcal meningitis [53]. In *E. coli*, it has been evidenced that OmpA^+^*E. coli* can induce ICAM-1 expression in hBMECs by activating NF-κB signaling [54] and that the IbeA^+^*E. coli* K1 strain can also induce activation and nuclear translocation of NF-κB in hBMECs [55]. In the current study, by western blotting, we also showed that the NF-κB pathway was activated more in hBMECs infected by meningitic strains PCN033 and RS218 compared with that by HB101 infection, where the phosphorylation of p65 and degradation of IκBα were compared, as well as with the immunofluorescence experiments that showed the nuclear translocation of p65. Not unexpectedly, treating hBMECs with the NF-κB inhibitor BAY11-7082 significantly attenuated those cytokines induction during meningitic *E. coli* infection, suggesting that NF-κB signaling works potently in mediating the neuroinflammatory response.

Likewise, we found that the effects of MAPK signaling were similarly associated with both PCN033 and RS218 infection of hBMECs. MAPK signaling cascades actually involve three major pathways: JNK (which acts as mediator of extracellular stress responses), ERK1/2 (which mediates proliferative stimuli), and p38 (which is also involved in mediating extracellular stress responses, particularly by regulating cytokine expression) [56]. Our IPA network analysis indicated the involvement of ERK during infection with meningitic *E. coli* PCN033 and RS218, which is consistent with our previous finding that MAPK/ERK signaling is involved in infection and mediates the induction of VEGFA and Snail-1 by the meningitic strain PCN033 [5]; however, via western blotting we showed the activation of all these three signaling molecules in response to PCN033 and RS218 infection. Also, by using specific inhibitors against ERK1/2, p38, and JNK, we observed that inhibition of all three MAPK pathways significantly decreased the infection-induced upregulation of proinflammatory cytokines IL-6, IL-8, IL-Iβ, and TNF-α. Therefore, collectively these data largely support the viewpoint that all three major MAPK signaling pathways play potent roles in meningitic *E. coli* infection and induce neuroinflammatory responses.

## Conclusions

In our study, using the iTRAQ proteomics approach, we compared and analyzed the DEPs in hBMECs infected with meningitic or non-meningitic *E. coli* strains. Twelve DEPs were identified as the commonly responding proteins in hBMECs upon infection with meningitic *E. coli* strains PCN033 and RS218, except for only one cellular protein shared by both meningitic and non-meningitic strains. Our data revealed MIF to be an important contributor to meningitic *E. coli*-induced cytokine production and tight junction disruption, while also showing that the NF-κB and MAPK signaling pathways are involved in the infection process. Comparing and profiling these differential cellular proteins in hBMECs in response to meningitic *E. coli* strains should open up further research on host responses against meningitic strains and help with the development of more targets for better prevention and therapeutic control of *E. coli* meningitis.

## Additional files

Additional file 1: **Table S1.** Protein profile of HB101-infected hBMECs. (XLSX 287 kb)

Additional file 2: **Table S2.** Protein profile of PCN033-infected hBMECs. (XLSX 290 kb)

Additional file 3: **Table S3.** Protein profile of RS218-infected hBMECs. (XLSX 289 kb)

Additional file 4: **Table S4.** GO term annotation of DEPs. (DOCX 15 kb)

Additional file 5: **Table S5.** Ingenuity Canonical Pathways of HB101-infected group. (XLSX 71 kb)

Additional file 6: **Table S6.** Ingenuity Canonical Pathways of PCN033-infected group. (XLSX 70 kb)

Additional file 7: **Table S7.** Ingenuity Canonical Pathways of RS218-infected group. (XLSX 71 kb)

Additional file 8: **Table S8.** The potential networks in HB101-infected group. (XLSX 70 kb)

Additional file 9: **Table S9.** The potential networks in PCN033-infected group. (XLSX 72 kb)

Additional file 10: **Table S10.** The potential networks in RS218-infected group. (XLSX 71 kb)

## Abbreviations

BBB: Blood-brain barrier
BMECs: Brain microvascular endothelial cells
CNS: Central nervous system
CSF: Cerebrospinal fluid
DEPs: Differentially expressed proteins
DMD: Dystrophin
*E. coli*: *Escherichia coli*
ECIS: Electric cell-substrate impedance sensing
EGFR: Epidermal growth factor receptor
ERK1/2: Extracellular signal-regulated kinases 1 and 2
ExPEC: Extraintestinal pathogenic *Escherichia coli*
GO: Gene Ontology
ICAM-1: Intercellular adhesion molecule-1
IL-1β: Interleukin 1 beta
IL-8: Interleukin-8
IPA: Ingenuity Pathways Analysis
ISO-1: (S, R)-3-(4-Hydroxyphenyl)-4, 5-dihydro-5-isoxazole acetic acid methyl ester DAPI 4′-6-Diamidino-2-phenylindole
iTRAQ: Isobaric tags for relative and absolute quantification
JNK: c-Jun N-terminal kinase
KEGG: Kyoto encyclopedia of genes and genomes
LC-MS/MS: Liquid chromatography tandem mass spectrometry
LGMN: Legumain
MAPK: Mitogen-activated protein kinase
MIF: Macrophage migration inhibitory factor
NF-κB: Nuclear factor-κB
NOS: Nitric oxide synthase
PI3K: Phosphatidylinositol 3-kinase
S1P: Sphingosine-1-phosphate
SCX: Strong cation exchange chromatography
TBPL1: TATA box-binding protein-like protein 1
TEAB: Tetraethyl-ammonium bromide
TEER: Trans-endothelial electric resistance
TNF-α: Tumor necrosis factor-alpha
VEGFA: Vascular endothelial growth factor A
ZO-1: Zonula occludens-1, IL-6, interleukin-6
