# Supplementary material for: New insights into meningitic Escherichia coli infection of brain microvascular endothelial cells from quantitative proteomics analysis
Source: J Neuroinflammation. 2018 Oct 19;15:291. doi: 10.1186/s12974-018-1325-z (PMC6195690; doi:10.1186/s12974-018-1325-z)
Supplement: Supplementary file 4 — Table S4. GO term annotation of DEPs. (DOCX 15 kb) [file 12974_2018_1325_MOESM4_ESM.docx]

**Supplemental. Table 4.** **GO term annotation of DEPs**

| Category name | Accession | Genes | | |
| --- | --- | --- | --- | --- |
|  |  | HB101 | PCN033 | RS218 |
| transporter activity | GO:0005215 | 0 | 1 | 0 |
| structural molecule activity | GO:0005198 | 1 | 6 | 5 |
| receptor activity | GO:0004872 | 0 | 0 | 2 |
| catalytic activity | GO:0003824 | 3 | 11 | 9 |
| binding | GO:0005488 | 2 | 22 | 17 |
| reproduction | GO:0000003 | 0 | 0 | 2 |
| multicellular organismal process | GO:0032501 | 1 | 1 | 2 |
| metabolic process | GO:0008152 | 5 | 29 | 22 |
| localization | GO:0051179 | 1 | 11 | 4 |
| immune system process | GO:0002376 | 0 | 1 | 1 |
| developmental process | GO:0032502 | 0 | 4 | 3 |
| cellular process | GO:0009987 | 3 | 31 | 21 |
| cellular component organization or biogenesis | GO:0071840 | 1 | 13 | 8 |
| biological regulation | GO:0065007 | 1 | 1 | 0 |
| biological adhesion | GO:0022610 | 0 | 0 | 1 |
| organelle | GO:0043226 | 1 | 11 | 9 |
| membrane | GO:0043226 | 0 | 5 | 1 |
| macromolecular complex | GO:0032991 | 2 | 12 | 5 |
| extracellular region | GO:0005576 | 1 | 0 | 0 |
| cell part | GO:0044464 | 1 | 21 | 12 |
